# Microglia in the context of multiple sclerosis

**DOI:** 10.3389/fneur.2023.1157287

**Published:** 2023-06-09

**Authors:** Xue Zhang, Fang Chen, Mingyue Sun, Nan Wu, Bin Liu, Xiangming Yi, Ruli Ge, Xueli Fan

**Affiliations:** ^1^Department of Neurology, Binzhou Medical University Hospital, Binzhou, China; ^2^Institute for Metabolic and Neuropsychiatric Disorders, Binzhou Medical University Hospital, Binzhou, China

**Keywords:** multiple sclerosis, microglia, remyelination, demyelination, signal transduction

## Abstract

Multiple sclerosis (MS) is an inflammatory and neurodegenerative disease that commonly results in nontraumatic disability in young adults. The characteristic pathological hallmark of MS is damage to myelin, oligodendrocytes, and axons. Microglia provide continuous surveillance in the CNS microenvironment and initiate defensive mechanisms to protect CNS tissue. Additionally, microglia participate in neurogenesis, synaptic refinement, and myelin pruning through the expression and release of different signaling factors. Continuous activation of microglia has been implicated in neurodegenerative disorders. We first review the lifetime of microglia, including the origin, differentiation, development, and function of microglia. We then discuss microglia participate in the whole processes of remyelination and demyelination, microglial phenotypes in MS, and the NF-κB/PI3K-AKT signaling pathway in microglia. The damage to regulatory signaling pathways may change the homeostasis of microglia, which would accelerate the progression of MS.

## 1. Microglia

### 1.1. The origin of microglia

**Figure 1:**
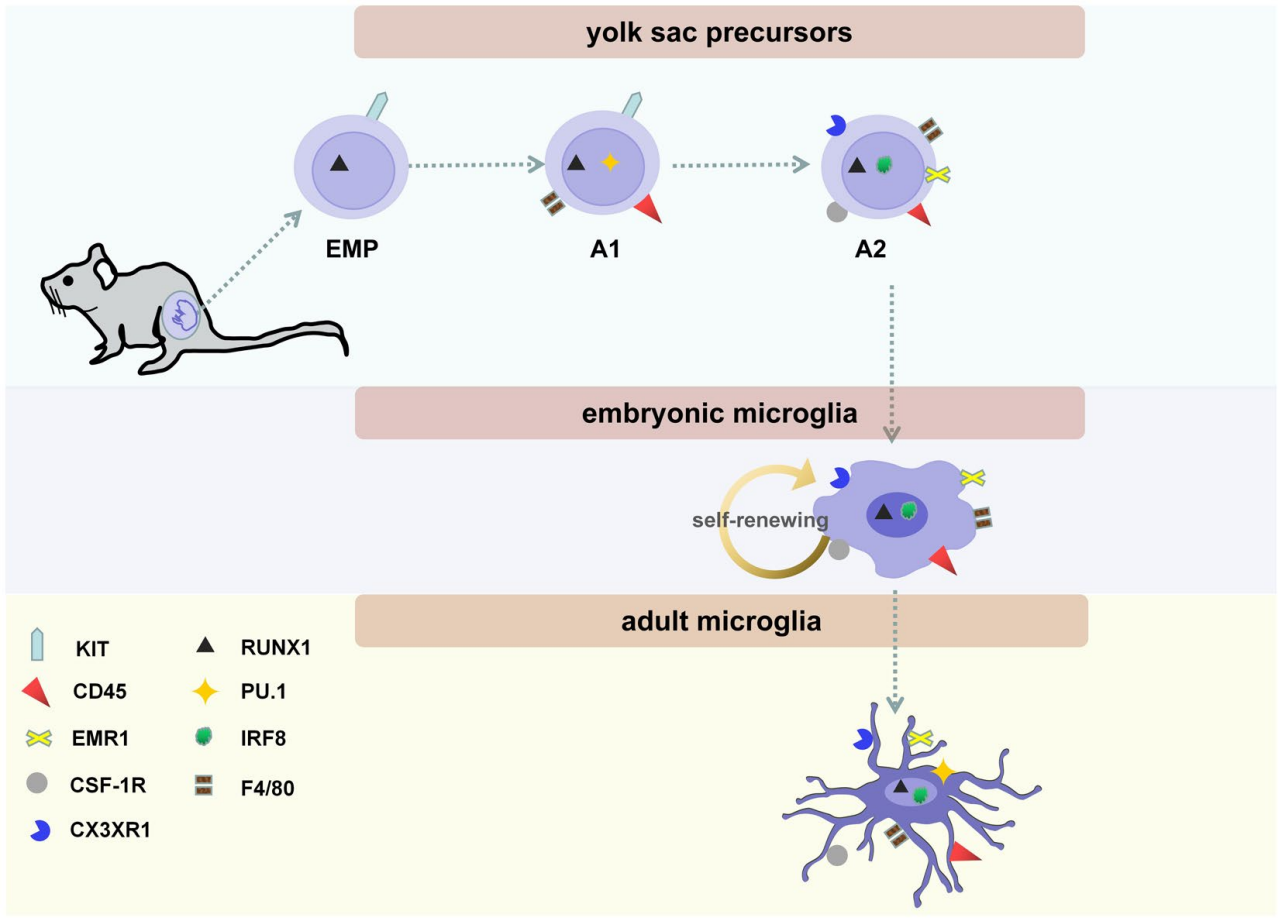
The three stages of microglial maturity. Microglial maturity at three stages: yolk sac precursors (including erythro-myeloid progenitors (EMPs), A1 and A2 cells), embryonic microglia, and adult microglia. On embryonic days (E)7.5–8.0, EMPs, which are KIT-positive, lineage marker-negative, can be found in the blood islands of the yolk sac. On E9.5, immature A1 and A2 cells are present on the surface of the developing brain. A1 cells begin to express CD antigen 45 (CD45); later, these cells develop into A2 cells. A2 cells begin expressing myeloid markers, including CX3C chemokine receptor 1 (CX3CR1, also called fractalkine receptor), colony-stimulating factor 1 receptor (CSF-1R, CD115), and emerin homolog 1 (EMR1, F4/80). In addition, the maturation of microglia is regulated by its endogenous transcription factors, including interferon regulatory factor 8 (IRF8), Runt-related transcription factor 1 (RUNX1), and PU.1. Embryonic microglia exhibit an amoeboid morphology without branching. Microglia are completely ramified on postnatal day (P)28 and maintain their ramified state until they become adult microglia.

Microglia were first discovered as a separate cell type by Pío del Río-Hortega in 1919. However, the exact origin of microglia remains unclear (1). Recently, an increasing number of studies have shown that microglia originate from yolk-sac progenitors during primitive hematopoiesis before the formation of bone marrow under homeostatic conditions (2) (Figure 1). Indeed, microglia were found in developing mouse embryos on embryonic day (E) 9.5 (3). On E9.5 of mouse embryonic development, immature A1 and A2 cells are localized on the surface of the developing brain. A1 cells began to express CD antigen 45 (CD45). During this period, myeloid markers are usually not expressed. Later, A1 cells further develop into A2 cells (4). Then, A2 cells migrate into the brain and begin expressing myeloid markers. A2 cells travel through the cerebral pial into the brain (5). It is presumed that many factors are involved in the process of microglial migration. No signaling molecule that can completely block microglia from entering the brain has been found (6).

### 1.2. The differentiation and development of microglia

In particular tissues, macrophages differentiate into different subtypes, such as Langerhans cells in the skin, alveolar macrophages, Kupffer cells, and microglia in the brain. Although these macrophages express many shared myeloid- and macrophage-specific markers, each tissue-resident population of macrophages have a distinct gene expression profile (7, 8). Microglia display specific transcriptomes and epigenomes, including transforming growth factor-beta (TGF-β), Spi-1 Proto-Oncogene (PU.1), interferon regulatory factor 8 (IRF8), and transmembrane protein 119 (Tmem119).

TGF-β1 signaling is indispensable for microglial survival. It is believed that the main downstream effectors of TGF-β signaling are small mothers against decapentaplegics (SMADs). In addition to controlling the activation state of microglia, TGF-β1 also maintains mature microglial homeostasis (9). For example, in TGF-β1-deficient mice, adult microglia fail to exhibit typical features, carry out their functions, and cannot survive (10). Other researchers have found that TGF-β1^−/−^ mice not only exhibit neurological defects and marked microglial alterations but also do not show the microglia-enriched gene signature (11, 12).

PU.1 is the most abundantly expressed erythroblast transformation-specific (ETS) transcription factor in microglia in both mice and humans (13). PU.1 expression is correlated with microglial development and function. Stimulus-dependent transcription factors (SDTFs) can induce the activation of enhancer profiles, which mediate the response of microglia to injury (14). According to recent studies, the knockdown of PU.1 makes microglia more susceptible to death (15). In addition, microglial development is impaired in mice lacking PU.1 (16).

IRF8 is a heterodimeric partner of PU.1 that can also regulate the transcriptional programming governing microglial development (3). Studies have proven the existence of a positive feedback loop between PU.1 and IRF8. IRF8 and PU.1 directly target reciprocal gene transcription (17). PU.1 mediates IRF8 expression by acting directly on the IRF8 gene locus. Similarly, IRF8 regulates PU.1 expression through one of the upstream regulatory elements (URE) of the PU.1 locus. During microglial activation, the abovementioned positive feedback loops sustain high expression of PU.1 and IRF8.

Tmem119 is also a specific marker of microglia via analyzing microglial transcriptome datasets in the physiological CNS (18). Unlike other molecules, the function of Tmem119 is still unknown (19). Tmem119 was also found to be expressed in lymph nodes, skeletal muscle, and brown adipose tissue (20, 21). Beyond that, Tmem119 could not label all microglia (20). Microglia could attenuate the expression of Tmem119 under pathological conditions (19). Hence, there are some limitations to using Tmem 119.

### 1.3. The function of microglia in the healthy brain

Microglia have attracted increasing attention because of their diverse functions. Microglia in the developing CNS and early postnatal CNS, together with microglia in the adult brain, represent various functional entities. Microglia not only guide neurons and axons to form prenatal circuits but also prune synapses and regulate synaptic plasticity. Moreover, microglia can present antigens and participate in the process of myelination and myelin pruning. Hence, microglia play a crucial role in maintaining the homeostasis of the developing brain and adult brain.

#### 1.3.1. Guidance and support

During prenatal development, microglia are located at the crossroads of significant neuronal migratory routes and axonal tract pathways, where they guide neurons and axons in forming prenatal circuits (22). Microglia participate in neurogenesis through the expression and release of signaling factors that impact the development and health of neurons, phagocytosing neural progenitor cells (NPCs) in the brain. Microglia also phagocytose growing axons during early brain development to regulate their growth, which helps shape new brain structures (6).

#### 1.3.2. Synaptic pruning

The classical complement cascade, which involves C3, C1q, and CR3 reside, is known to play a key role in synaptic pruning in the brain. C1q activates the cascade, and then C3 coats the offenders and attracts receptor 3 (CR3; a heterodimer of CD11b) to tag “weaker” synapses. Depletion of any critical players in this cascade (C1q, C3, or CR3) increases the number of synapses (23). Synaptic pruning is also regulated by the CX3CL1-CX3CR1 signaling pathway. In CX3CR1-deficient mice, synaptic pruning is impaired to some extent (24).

#### 1.3.3. Synaptic plasticity

Microglia mediate synaptic refinement in the postnatal brain (25). Microglia-induced synaptic plasticity is dependent on BDNF secretion, which increases the phosphorylation of neuronal tropomyosin kinase receptor type B (TrkB) (26). BDNF-TrkB signaling is involved in long-term potentiation (LTP) induction, which is the principal mechanism of synaptic plasticity (27). Changes in the number of synapses can also cause changes in synaptic plasticity, so synaptic pruning is also crucial for the regulation of synaptic plasticity (28).

#### 1.3.4. Antigen presentation

It has been suggested that microglia are the primary antigen-presenting cells (APCs) in the CNS (29). Major histocompatibility complex (MHC) molecules are expressed at low levels in microglia (30). Under steady state conditions, MHC molecules are responsible for antigen presentation. Adult microglia also express genes associated with surveillance and the immune response, so they can constantly monitor the external environment. After stimulation, microglia can express *de novo* MHC and are able to develop into APCs (31).

#### 1.3.5. Myelination and myelin pruning

In addition to these important functions, microglia secrete growth factors and support oligodendrocyte progenitor cells (OPC) and oligodendrocytes. Therefore, microglia can promote myelination under steady-state conditions. Under pathological conditions (for example, in MS), microglia also participate in the process of remyelination and demyelination. During the normal aging process, they also phagocytose excess myelin sheaths, which is called myelin pruning (23).

## 2. Microglia in multiple sclerosis

MS is the most common nontraumatic disabling disease among young adults (32). Additionally, MS is an inflammatory and neurodegenerative disease of the brain and spinal cord, and the characteristic pathological hallmark of MS is the loss of myelin, oligodendrocytes, and axons (33). Relapse remitting MS (RRMS), which accounts for 80–90% of MS cases, begins with episodes of neurological disorders and then partial or complete remission (34). Upon aging, patients with RRMS develop secondary progressive multiple sclerosis (SPMS) with fewer remissions and increasing clinical deterioration (35). Only 15% of MS patients have primary progressive MS (PPMS), which involves a steady and progressive loss of neurological function from the onset of the disease (36, 37). Progressive relapsing MS (PRMS) is the most uncommon form of MS, affecting approximately 5% of MS patients, and is characterized by a steady decline in health with unexpected spikes of deterioration and recovery (38). Although the clinical characteristics of MS are clear, the pathology of MS is a dynamic and continuous process (39).

Microglia provide continuous surveillance of the CNS microenvironment and initiate defense mechanisms to protect CNS tissue. Oligodendrocytes, the myelinating cells of the CNS (40), are often targets of autoimmune pathology during MS progression (41). There is emerging evidence that microglia actively contribute to inflammation that directly and indirectly contributes to neurodegeneration. Microglia are highly dynamic, so they can recognize changes in the cerebral parenchyma with their highly motile processes (2, 42). Microglia often keep a specific ramified morphology in healthy adult brain tissue. As soon as signs of injury, such as inflammation or tissue injury, are detected, microglia rapidly move toward the lesion site and transition into an activated state (43–45). Continued activation of microglia drives neuroinflammation and neurodegeneration (46).

### 2.1. Demyelination and myelin regeneration in MS: microglia and OPC

Myelin sheaths are generated by oligodendrocytes and wrap around axons (47, 48). As mentioned above, the characteristic pathological hallmark of MS is the loss of myelin, oligodendrocytes, and axons. Remyelination refers to the restoration of the myelin sheath around denuded axons (49) and is activated by the recruitment and differentiation of OPC (50). After migrating to the CNS, OPC adopt a premyelinating phenotype (preoligodendrocyes) and can wrap around axons. However, preoligodendrocytes are unable to form mature myelin. The expression of myelin basic protein (MBP) and myelin oligodendrocyte glycoprotein (MOG) indicates the development of mature oligodendrocytes (51). Mature oligodendrocytes contact and wrap neuronal axons with myelin while being connected to surviving myelin sheaths (52). While a substantial number of OPC are found in and around MS lesions, OPC recruitment and the differentiation of OPC into mature myelinating oligodendrocytes are impaired (53). Remyelination becomes less efficient as patients grow older and lesions become more chronic (50, 54). According to a number of studies, the development of OPC and oligodendrocytes is affected by protein products expressed by microglia (6, 55). For instance, microglia-conditioned medium increases the expression of platelet-derived growth factor (PDGF), vascular endothelial growth factor (VEGF), and insulin-like growth factor 1 (IGF-1), which promote the survival and maturation of OPC.

**Figure 2:**
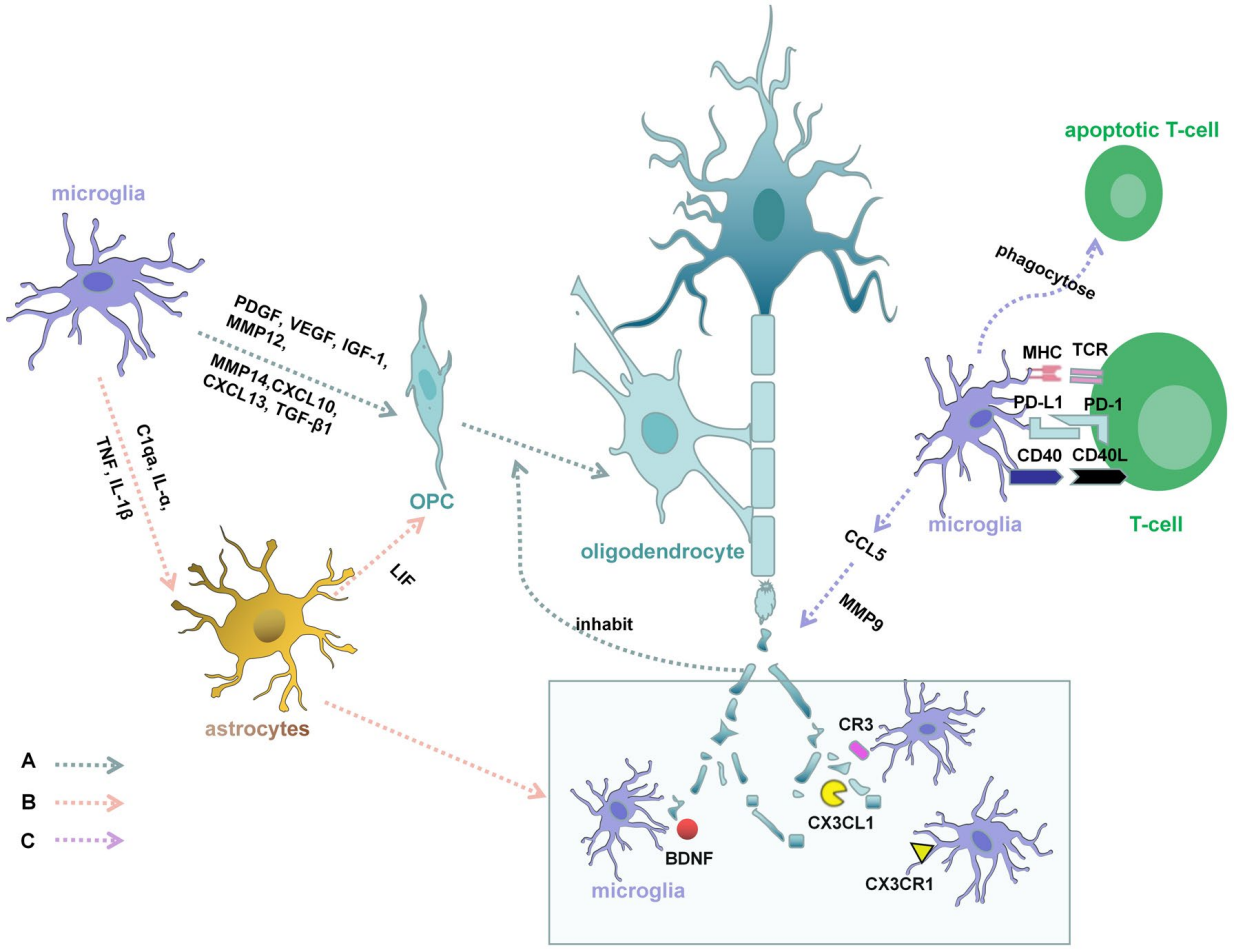
Demyelination and myelin regeneration in MS. A: Microglia can express platelet-derived growth factor (PDGF), vascular endothelial growth factor (VEGF), insulin-like growth factor 1 (IGF-1), CXCL10, CXCL3, TGF-β1, MMP12, and MMP14, which promote the survival and maturation of OPC and supporting oligodendrocyte remyelination. Myelin debris inhibits OPC differentiation, which markedly affects remyelination. From the onset of demyelination, microglia provide a favorable environment for clearing debris. The brain-derived neurotrophic factor (BDNF), CX3CR1, CX3CL1, and C3R are involved in this process. B: C1qa, interleukin-ɑ (IL-ɑ), and Tumor necrosis factor (TNF) secreted by microglia, which could increase the reactivity of astrocytes. Interleukin-1β (IL-1β) secreted by microglia, can activate astrocytes and allow the astrocytes to produce leukemia inhibitory factor (LIF). LIF can promote the differentiation of OPC into mature myelinating oligodendrocytes and relieve demyelination. Microglia are also recruited by astrocytes to the demyelinating lesions, which ensures the smooth process of phagocytosis. C: Encephalitogenic T cells might produce cytokines that directly activate microglia. Activated microglia are the principal source of CC and CXC-chemokines, such as CCL5. CCL5 promotes the activation and secretion of metalloproteinase-9 (MMP-9) in human microglia, which is in connection with the degradation of myelin proteins. Microglia also have a great ability to phagocytose apoptotic encephalitogenic T cells, which can influence the chemoattractive milieu.

Microglia are involved in both the de and remyelination phases of MS (Figure 2). From the onset of demyelination, microglia provide a favorable environment for myelin regeneration (56), recruiting new OPC, providing trophic support, repairing damaged tissue, and clearing debris (57). In the surrounding MS lesions, microglia express Semaphorin-3F (SEMA3F), which can attract OPC to damaged areas (58). Apart from that, studies have also found that microglia express 6,200 genes, including the upregulated genes Lrp1, Calr, CXCL10, CXCL13, Pdgfa, Pdgfb, Vegfa, Vegfb, TGF-β1, MMP12, and MMP14, in the cuprizone-induced demyelination model. These upregulated genes are involved in phagocytosing apoptotic cells and debris, recruiting OPC, and supporting oligodendrocyte remyelination, differentiation, and tissue remodeling (59). Microglia have been proven to be fundamental for remyelination (60). To evaluate the effect of microglia on myelination, researchers examined the myelination of oligodendrocytes in larvae lacking microglia. IRF8 and CSF-1R are necessary for microglial development (3, 61). To eliminate microglia in the early stages of development, researchers used an antisense morpholino oligonucleotide designed to block the translation of IRF8 messenger RNA and a CSF-1R inhibitor that was used previously in larval zebrafish (62). Under these conditions, oligodendrocytes exhibited more sheaths (23). Myelin debris inhibits OPC differentiation, which markedly affects remyelination (63). In CX3CR1 gene knockout mice, phagocytosis of microglia is dramatically reduced after treatment with cuprizone, resulting in the persistence of myelin debris and inhibition of proper remyelination due to impaired OPC recruitment (60, 64, 65). Phagocytosis of myelin debris by microglia is essential for the initiation of lesion repair (66). Triggering receptor expressed on myeloid cells (TREM2), which is expressed on the cell surface and binds polyanions, thus activating downstream signaling cascades through the adapter DAP12 (also called TYRO protein tyrosine kinase binding protein) (67), has been found to be related to phagocytosis (68). In Trem2−/− mice, clearance of myelin debris and axonal support are impaired, the number of oligodendrocytes is increased, and inflammatory mediators are expressed after long-term cuprizone treatment, resulting in persistent demyelination (69). Moreover, myelin interacts with microglial MER proto-oncogene tyrosine kinase (MERTK). MERTK is indispensable for myelin phagocytosis (65). *In vitro* studies have shown that microglia stimulated with TGF-β express increased MERTK receptors, which have a good ability to clear myelin debris (70). A series of studies revealed the multiple mechanisms by which microglia exert beneficial effects on OPC survival and maturation (71).

### 2.2. Demyelination and myelin regeneration in MS: microglia and astrocytes

Classically, active MS lesions are highly inflammatory, showing infiltration of many lymphocytes, including CD8+ T cells, CD20+ B cells, macrophages, and CD4+ T cells (33, 72). In addition, resident cells of the CNS, including activated microglia and reactive astrocytes, are present in MS lesions (73). These infiltrating cells produce a large number of cytokines and other inflammatory factors that contribute to myelin destruction and axonal degradation. The degree of myelin regeneration depends on the severity of the disease. Microglia have an effect on the function of astrocytes (Figure 2). Researchers have discovered that microglia are indispensable for the activation of astrocytes by using cuprizone (CPZ)-induced mouse models of demyelination and remyelination (74). In mice that knock out cytokines secreted by microglia, including C1qa, interleukin-ɑ (IL-ɑ), and Tumor necrosis factor (TNF), the reactivity of astrocytes was reduced (75). Interleukin-1β (IL-1β) secreted by microglia, can activate astrocytes and allow the astrocytes to produce leukemia inhibitory factor (LIF) (76). LIF can promote the differentiation of OPC into mature myelinating oligodendrocytes and relieve demyelination in an animal model of multiple sclerosis (77). In further experiments, researchers have found astrocytes are also involved in the process of myelin debris removal (75). Microglia are recruited by astrocytes to the demyelinating lesions, which ensures the smooth process of phagocytosis (78). When the process is broken, OPC do not mature properly, resulting in the failure of remyelination. In MS, microglia participate in the whole processes of remyelination and demyelination (Figure 2).

### 2.3. Microglia and T cells interactions in MS

T lymphocytes are the basic organizers of most autoimmune responses, including MS (Figure 2). In MS, the encephalitogenic T cells might produce cytokines that directly activate microglia or through inducing the cerebral tissue damage indirectly activate microglia (79). Eugene D. Ponomarev first discovered that microglial activation in the CNS takes precedence over the appearance of neurological impairment symptoms in EAE and the peripheral macrophage cells flow over into the CNS, which further explains the activation of encephalitogenic T cells are necessary to the EAE onset (80). Activated microglia are the principal source of CC and CXC-chemokines, such as CCL2, CCL3, CCL4, CCL5, CCL12, and CX3CL1 (81). All of chemokines’ biological effects are performed by binding ligands to receptors. Chemokines play the main role in immunity and inflammation in MS. They attract the migration of pathogenic cells, interfering immune regulation of T-lymphocytes (82). Moreover, CCL5 promotes the activation and secretion of metalloproteinase-9 (MMP-9) in human microglia (83). MMP-9 is not only involved in the process of leukocyte extravasation but also in connection with the degradation of myelin proteins (84). Microglia also have a great ability to phagocytose apoptotic cells in the CNS, including encephalitogenic T cells, which can influence the chemoattractive milieu (85). Therefore, microglia may further disturb encephalitogenic T cells recruitment.

### 2.4. Paramagnetic rim lesions in MS

In MS, the white matter lesions are classified into active, inactive, and remyelinated lesions according to the distribution of inflammatory cells and the severity of demyelination (86). Slowly expanding chronic active lesions (CALs) mainly happen to those who occur 10 years after onset and CALs were seen more often in progressive MS than in relapsing disease (87). The paramagnetic rim lesions (PRLs) have been considered diagnostic MS biomarkers, which identify the CALs (88). Histological analysis has shown that PRLs correspond with iron-laden active microglia at the edges of CALs and indicate compartmentalized inflammation (89–91). When oligodendrocytes and myelin were damaged, microglia could phagocytose the iron released in the cells and the iron-laden microglia occurred at the MS lesions (92). Neuroimaging studies have revealed that the patients with progressive disease, compared to the patients with relapsing disease, have more rate of PRLs (93, 94). The clinical correlation about PRLs and more serious neurological impairments, is a more complex course. PRLs tend to the extent of involved lesions expanding 2 percent annually, according to a recent study on a new study on PRLs at 3-T MRI (93).

### 2.5. Microglial phenotypes in multiple sclerosis

As noted above, microglia have different functions in the embryonic, early postnatal, and adult stages and are multifunctional cells. Functional plasticity allows activated microglia to be polarized to either the M1 (pro-inflammatory) phenotype or the M2 (anti-inflammatory) phenotype (95). M1 microglia secrete pro-inflammatory mediators and oxidative compounds, including IL-1, IL-12, IL-23, Iba-1, CD68, IL-1β, TNF-α, and nitric oxide (NO). Additionally, MHC class II, costimulatory molecules, Fc receptors, and integrins are expressed in M1 microglia. Eventually, M1 microglia become cytotoxic and promote inflammation (67, 96, 97). In contrast, M2 microglia can promote tissue repair by releasing anti-inflammatory mediators such as IL-4, IL-10, and IL-13 and upregulating the expression of the M2 markers CD206 and arginase 1 (Arg1) (98). M2 microglia also secrete growth factors and neurotrophic factors, such as IGF-I, CSF1, nerve growth factor (NGF), BDNF, neurotrophin (NT)4/5, and glial cell-derived neurotrophic factor (GDNF).

Microglia are able to switch between the pro-inflammatory and anti-inflammatory states to maintain tissue homeostasis in response to the environment (99). In the experimental autoimmune encephalomyelitis (EAE) model of multiple sclerosis, both M1 and M2 microglia play vital roles in remyelination (51). In early demyelination, the M1 phenotype predominates, while M2 microglia are more abundant in later stages. It has been discovered that M2 microglia drive oligodendrocytes differentiation and remyelination. When remyelination starts, M1 microglia obviously transform into anti-inflammatory microglia. When remyelination is efficient, the M2 number is increased. Furthermore, M2 microglia-conditioned medium promotes the differentiation of oligodendrocytes, while the M1-conditioned medium inhibits this process (100). Despite this, microglial activation is not a simple dichotomy but is part of a spectrum of functional states (3). Emerging evidence suggests that the concept of M1/M2 microglia polarization may be outdated. In fact, transcriptome studies have shown that microglial activation is varied and context dependent (22).

Furthermore, according to their morphological characteristics, microglia can be divided into four types. These include what are known as ramified (micro somata but long ramifications), amoeboid (ramifications shorter, the cell somata larger), and phagocytic (no ramifications, just a rounded cell) (101). And dystrophic microglia are another cellular phenotype of microglia, which was considered to be one form of aging in microglia (102). Dystrophic microglia are rich among old people, and more and more recent studies discovered it also has been relationship to neurodegenerative diseases (103), such as MS. The morphology of dystrophic microglia is very different from hypertrophic microglia. They are characterized by fragmentation of cytoplasm, serious deformities of mitochondria, and distal branches becoming thinner (104). Research has suggested while M2 microglia play a protective role for CNS, finally they can convert to a dystrophic state (105). Dystrophic microglia produce more inflammatory mediators and loss the function of neuroprotection, which cause the disease to become worse (106).

As alluded to above, microglial activation is not a simple dichotomy. The whole-genome transcriptomics and unbiased proteomics studies have clarified problems associated with the microglial polarization response. The mixed phenotypes (intermediate phenotypes) have M1 and M2 markers at the same time, which occurs mainly in aging and various pathologic conditions, meaning M1 and M2 represent part of a spectrum of various activation phenotypes (107, 108). Even though an increasing number of people have realized that such a simplistic dichotomy (M1/M2) could not completely represent the complex phenotypes of microglia *in vivo*, this classification is important to the development of PET tracer (109). Under the circumstances, we interpret the M1 (pro-inflammatory) in connection with neurotoxic functions and the M2 (anti-inflammatory) involved in recovery and reduce harmful effects.

### 2.6. The PI3K-AKT pathway in microglia may be involved in MS

In MS, signal transduction pathway abnormalities in microglia may be the main causes of microglial activation and exacerbation of neuroimmune responses. Studies have demonstrated that the phosphatidylinositol 3-kinase (PI3K) and protein kinase B (AKT, also commonly known as protein kinase B (PKB)) signaling pathways play a crucial role in the modulating microglial activity and inflammatory responses (110). The PI3K-AKT pathway regulates cellular activities such as neuronal cell proliferation, migration, and plasticity. PI3K-AKT signaling in the brain seems to be closely related to microglial activity and activation. An increasing number of studies have shown that aberrant PI3K-AKT signaling participates in neurodegeneration and neuroinflammatory diseases (111).

Microglia express many key regulatory receptors that activate the PI3K-Akt pathway (112). As one of the first lines of immune defense, microglia express a number of different toll-like receptors (TLRs), including TLR2, TLR3, TLR4, TLR5, TLR7, and TLR9, on their surface (41). Among TLRs, TLR4 is an important receptor that mediates the inflammatory response and can interact with various immune stimulants. TLR4, which interacts with lipopolysaccharide (LPS), delivers downstream signals through myeloid differentiation primary response 88 (MyD88). Then, MyD88 phosphorylates tyrosine residues. Several studies have provided evidence that PI3K-AKT signaling is required for LPS-TLR4-dependent activation of microglia (113). Increased expression of TLR4 or constant TLR4 stimulation can lead to the subsequent activation of PI3K-AKT. PI3K is recruited via its p85 domain, which leads to downstream signal activation (114, 115). In this setting, microglia are constantly activated, and neuroinflammation is perpetuated. Increased TLR4 expression on microglia suppresses their polarization toward the anti-inflammatory phenotype and simultaneously prolongs the microglia-mediated proinflammatory response (116). In the brain, CSF-1R is primarily expressed by microglia (117). Contact of the CSF-1R receptor with microglia leads to oligomerization and the phosphorylation of its tyrosine residues, which are indispensable for PI3K recruitment (118). When expressed at high levels, CSF-1R and CSF-1 are considered mediators of demyelination in progressive MS, which exacerbates neuroinflammation due to the survival and constant proliferation of microglia (119). Furthermore, CX3CR1 activates the PI3K-AKT signaling pathway in a dose-dependent manner (51). CX3CR1 is a gene that is highly expressed in microglia. In summary, abnormal expression of TLR-4, CSF-1R, and CX3CR1 leads to aberrant PI3K-AKT signaling and impairs brain development, thus promoting the onset of neurodegenerative diseases such as MS.

Currently, the role of PI3K-AKT signaling in neuroimmunology is still incompletely understood. The PI3K-AKT pathway regulates microglia in response to various extracellular stimuli. It plays an important role in activating microglia to produce proinflammatory mediators after stimulation (110). Researchers have found the lipophilic amino alcohol 4b can attenuate the pathogenesis of EAE by inhibiting the PI3K-AKT pathway (120). The emodin also has therapeutic effect on EAE mice, which can down-regulate the expression of phosphorylated (p)-PI3K, p-Akt and further inhibit microglia activation and inflammation (121). However, the PI3K-AKT pathway may also play a neuroprotective role in different diseases. Researchers have also confirmed that PI3K-AKT pathway activation is important for oligodendrocytes survival and axonal myelination in the EAE model (122).

### 2.7. The NF-κB signaling pathway in microglia may be involved in MS

Another signaling pathway associated with MS is the nuclear factor-κB (NF-κB) signaling pathway. The NF-κB, expressed by many cells such as microglia, neurons, and astrocytes, plays a strong part in inflammation and immunity (123). The NF-κB signaling system is controlled by three interacting parts: NF-κB dimers, an inhibitor of NF-κB (IκB) regulators, and IkappaB kinase (IKK) complexes (124).

The activation of NF-κB is regulated by the canonical and non-canonical pathways. The canonical pathway can be activated by all kinds of stimuli, including cytokines, a number of pathogens, and different types of stress, which regulate proinflammatory gene expression (125). The non-canonical pathway was mainly activated by the TNF receptor superfamily members (126). This pathway contributed to maintaining immune homeostasis by participating in the development of the lymphoid tissues and various immune cells under healthy physiological conditions (126). In microglia, the pathway can be activated Fas ligand after the combination with TNF-α (126).

In MS, the activation of NF-κB in microglia is a reaction to injury to the CNS. Activated NF-κB leads to a cascade of signaling events, including the production of IL-1, which promotes microglia generating more proinflammatory cytokines, nitric oxide (NO), and neurotoxic reactive oxygen species (127). These cause a prominent toxic effect on the nervous system and exacerbate neuronal degeneration. Under the stimulation of pathology, the largest group of activated NF-κB form in the canonical pathway is the RelA: p50 (p65: p50) heterodimer, which plays a significant role in chronic inflammatory disease and neurodegenerative pathologies such as MS (125). Some compounds widespread in nature and some kinds of suppressors of cytokine signaling, perform a beneficial role in the treatment of EAE by downregulating NF-κB p65 signaling. Belinostat, the inhibitor of histone deacetylase, can meliorate symptoms of EAE through the downregulation of NF-κB p65 protein expression (128). Matrine (MAT), naturally present in *Sophora flavescens*, also alleviates the condition of EAE. MAT downregulates NF-κB p65 Phosphorylation, which also happened during the treatment courses of EAE (129). In LPS-induced microglia, the high-density lipoprotein (HDL) could significantly reduce the expression of TLR4 and NF-κB p65 (130). And in EAE, HDL could alleviate the soakage of inflammation cells in the spinal cord and brain, reducing the ratio of M1 microglia (130). Microglia also influence the development of EAE by regulating the noncanonical NF-κB pathway. Research showed that the noncanonical NF-kB pathway in microglia can interact with T cell-derived cytokine, which can accelerate the process of MS (131). Therefore, it has been a long-awaited goal to treat MS by directly targeting NF-κB pathway activation.

## 3. Conclusion

It is well known that remyelination failure is a significant challenge in the treatment of MS. Microglia, which are involved in myelin regeneration and play versatile roles in the pathogenesis of neuroinflammation and neurodegeneration, may be key therapeutic targets for diseases. Further studies are needed to better elucidate the precise roles of microglia in diseases and identify new therapeutic options that not only prevent new damage but also restore lost function.

## Author contributions

All authors listed have made a substantial, direct, and intellectual contribution to the work and approved it for publication.

## Funding

This work was supported by the National Natural Science Foundation of China (grant nos. 81701192 and 81901380), Shandong Provincial Natural Science Foundation, China (grant nos. ZR2017BH078 and ZR2017BC047), and Scientific Research Foundation of Binzhou Medical University (grant nos. BY2017KYQD15 and BY2016KYQD21).

## Conflict of interest

The authors declare that the research was conducted in the absence of any commercial or financial relationships that could be construed as a potential conflict of interest.

## Publisher’s note

All claims expressed in this article are solely those of the authors and do not necessarily represent those of their affiliated organizations, or those of the publisher, the editors and the reviewers. Any product that may be evaluated in this article, or claim that may be made by its manufacturer, is not guaranteed or endorsed by the publisher.
